# Dietary supplementation and the role of phytochemicals against the Alzheimer's disease: Focus on polyphenolic compounds

**DOI:** 10.1016/j.tjpad.2024.100004

**Published:** 2025-01-01

**Authors:** Rayees Ahmad Naik, Roshni Rajpoot, Raj Kumar Koiri, Rima Bhardwaj, Abdullah F. Aldairi, Ayman K. Johargy, Hani Faidah, Ahmad O. Babalghith, Ahmed Hjazi, Walaa F. Alsanie, Abdulhakeem S. Alamri, Majid Alhomrani, Abdulaziz Alsharif, Anastasiia Shkodina, Sandeep Kumar Singh

**Affiliations:** a Biochemistry Laboratory, Department of Zoology, Dr. Harisingh Gour Vishwavidyalaya Sagar, Madhya Pradesh, 470003, India; b Department of Chemistry Poona College, Savitribai Phule Pune University, Pune 411007, India; c Department of Clinical Laboratory Sciences, Faculty of Applied Medical Sciences, Umm Al-Qura University, Makkah, Saudi Arabia; d Department of Microbiology and Parasitology, Faculty of Medicine, Umm Al-Qura University, Makkah, Saudi Arabia.; e Department of Medical Genetics, Faculty of Medicine, Umm Al-Qura University, Makkah, Saudi Arabia; f Department of Medical Laboratory Sciences, College of Applied Medical Sciences, Prince Sattam bin Abdulaziz University, Al-Kharj 11942, Saudi Arabia; g Department of Clinical Laboratory Sciences, The faculty of Applied Medical Sciences, Taif University, Taif, Saudi Arabia; h Research Centre for Health Sciences, Deanship of Graduate Studies and Scientific Research, Taif University, Saudi Arabia; i Department of Neurological diseases, Poltava State Medical University, Poltava, 36000, Ukraine; j Indian Scientific Education and Technology Foundation, Lucknow, 226002, India

**Keywords:** Alzheimer's disease, Oxidative stress, Mitochondrial dysfunction, Phytochemical

## Abstract

Alzheimer's disease is a complicated, multifaceted, neurodegenerative illness that places an increasing strain on healthcare systems. Due to increasing malfunction and death of nerve cells, the person suffering from Alzheimer's disease (AD) slowly and steadily loses their memories, cognitive functions and even their personality. Although medications may temporarily enhance memory, there are currently no permanent therapies that can halt or cure this irreversible neurodegenerative process. Nonetheless, fast progress in comprehending the cellular and molecular abnormalities responsible for neuronal degeneration has increased confidence in the development of viable prevention and treatments. All FDA-approved anti-AD medications have merely symptomatic effects and cannot cure the illness. This necessitates the pursuit of alternate treatments. Accumulating data shows that systemic neuroinflammation, oxidative stress and associated mitochondrial dysfunction play crucial roles in the etiology of AD and precede its clinical presentation. Therefore, innovative therapeutic approaches targeting these pathophysiological components of Alzheimer's disease are being explored aggressively in the present scenario. Phytochemicals such as resveratrol, curcumin, quercetin, genistein and catechins are prospective therapies owing to their capacity to alter key AD pathogenetic pathways, such as oxidative stress, neuroinflammation, and mitochondrial dysfunction. The use of new phytochemical delivery strategies would certainly provide the possibility to solve several issues with standard anti-AD medicines. In this review, the roles of phytophenolic compound-based treatment strategies for AD are discussed.

## 1 Introduction

Dementia is a broad spectrum in neurodegenerative disorders characterized by a significant reduction in cognitive capacity that impairs daily activities. Alzheimer's disease (AD) is the most prevalent form of dementia, related to age. It causes chronic cognitive loss and prevalent neurodegeneration. The occurrence of genetically determined AD due to specific mutations is limited to 1-2 % of cases. Although therapeutic interventions for symptom management are at present accessible, AD remains incurable. An estimated fifty million individuals worldwide are afflicted with AD, and the disease continues to spread. (World Alzheimer's Report) [1]. AD is associated with a gradual decrease in both the autophagy-lysosomal pathway as well as the ubiquitin-proteasome system (UPS), both of which are essential catabolic reactions in eukaryotes. (ALP) [2]. Proteostasis is regulated by both the ALP and UPS pathways, which combine to form a single structure for protein stabilization. An accumulation of detrimental proteins within the cells can occur when the functionality of UPS and ALP is compromised; this, in turn, can accelerate the development of Alzheimer's disease [2]. Dysfunction in the crucial lysosomal pathway transmembrane protein (TMEM)175 is a defining characteristic of a number of neurodegenerative disorders, including Alzheimer's disease, Parkinson's disease, and Huntington's disease [3]. At this time, AD represents a substantial worldwide social and medical issue. The most prominent pathogenic features of Alzheimer's disease (AD) are the deposition of amyloid beta (Aβ) in the brain and the formation of neurofibrillary tangles (NFTs), which are distinguished by the excessive phosphorylation of tau proteins. [4]. Oxidative stress, neuroinflammation, mitochondrial dysfunction, and biometabolic imbalance are all potential consequences of the damage induced by Aβ and NFT. These effects can culminate in the dendritic spine reduction, synaptic loss, and neuronal demise. [5].

One extensively researched concept pertaining to the initiation and progression of Alzheimer's disease (AD) is the cholinergic hypothesis. This hypothesis, which was the initial idea proposed concerning the pathogenesis of AD, has garnered significant attention (Hampel et al., 2019). In people with Alzheimer's disease (AD), brain shows various pathological signs, such as shrinkage, synaptic loss, and reduced central neurotransmission, as well as the presence of histopathological markers. There is a general decline in the functioning of basal forebrain neurons [6]. At the onset of the illness, there is a decline in the population of cholinergic neurons located in the basal nucleus and entorhinal cortex. However, in the later stages of Alzheimer's disease, over 90 % of the cholinergic neurons in the basal nucleus experience loss [7]. Based on the cholinergic hypothesis, it is suggested that the dysfunctional or impaired operation of the cholinergic system might lead to a memory impairment in animal models, resembling Alzheimer's disease [8]. Based on this concept, the degeneration of cholinergic neurons located in the basal forebrain, together with the subsequent decline in central cholinergic transmission, is believed to be responsible for the manifestation of both psychological and non-cognitive characteristics observed in individuals diagnosed with AD. [9].

Because there are no effective therapies for Alzheimer's disease, innovative prevention techniques are being developed based on dietary modifications, vitamin and mineral supplements, healthy foods, and natural substances. Lately, there have been instances an increased focus on natural phytochemicals as potential alternative treatment agents for Alzheimer's disease [10]. The characterization of the therapeutic potential of natural chemical structures in neurodegenerative illnesses, such as Alzheimer's disease (AD), has become increasingly important due to recent advancements [11].

Phytochemicals are a potent substance found in plants, which are classified as secondary metabolites. They include a wide variety of chemical groups, including polyphenols, flavonoids, steroidal saponins, organosulphur compounds, and vitamins. They play crucial roles in development of plants, participating in significant physiological processes like as reproduction, symbiotic organizations, and interactions with other species and the environment. Plants generate phytochemicals (e.g., phenols, terpenes, and organosulfur) promote pigmentation, smells, and allergens that may protect the plant from internal (e.g., metabolic) and exterior (e.g., environmental) threats to its survival, including infectious agents, predatory animals, UV rays, as well as ROS and protein excessive expression, respectively. Humans appear to derive health benefits from consuming plants that produce these phytochemicals through the modulation of multiple biological mechanisms such as inflammatory reactions, death of neuronal cells (apoptosis), growth of neurons, neural communication, and activity of enzymes are involved [12]. Possible reasons for these impacts might involve an antioxidant and anti-allergic characteristics and regulation of Aβ levels and toxicity. Various pharmaceutical therapies for Alzheimer's disease are originating from conventional herbal treatments. An AChE blocker referred to as galantamine is obtained from daffodil plants, whereas an anti-inflammatory drug called aspirin is produced through the synthesis of salicylic acid, a polyphenol discovered in the outer bark of willow shrubs. Various phytochemicals are being studied over potential use in alleviating Alzheimer's disease. [13]. Furthermore, there has been a growing interest in investigating the purpose and processes of nutrition phytochemical compounds with respect to the role of the gastrointestinal microbiome. There is an expanding field of interest in examining the correlation between age-related amyloid genesis, proinflammatory microbial activities, and neurodegeneration [14]. The gastrointestinal microbiota also has been highly involved in the metabolism and biological activation of nutritional phytochemicals, in addition to its role in the pathogenesis of AD. Initial absorption of dietary phytochemicals has been shown to range from 5 to 10 %. Numerous microbiomes produced phenolic metabolites, according to recent investigations of their pharmacokinetic action, attained statistically significant volumes in the brain. [15].

Phytochemicals can be ingested through means other than the diet. For instance, it was hypothesized that tobacco use might supply a degree of defense against accumulation of Aβ and the progress of AD. This was largely attributable to postmortem examinations of the brains of Alzheimer's disease patients, which revealed that smokers had substantially reduced levels of Aβ in the entorhinal cortex. [16]. Conversely, Smoking is amyloid beta accumulation identified as a predictive factor for the onset of Alzheimer's disease (AD). in recent epidemiological researches [17]. Consistent consumption of fruits and vegetables containing a significant amount of bioactive phytochemical substances may reduce the possibility of acquiring AD in an integrated and synergistic manner, according to prior research. The apparent health-promoting properties of natural products as therapeutics for AD have been confirmed in multiple studies [18]. Furthermore, a variety of epidemiological studies have provided evidence regarding the correlation between dietary patterns and the occurrence of neurodegenerative diseases. A notable positive correlation has been proposed between the ingestion of foods abundant in polyphenolic phytochemicals and the prevention of specific neurological disorders, such as Alzheimer's disease. [12]. From a variety of natural substances that are gaining attention for having potential anti-AD characteristics, this study will concentrate on polyphenolic phytochemicals. These phytochemicals may exhibit anti-amyloidogenic, anti-oxidative, and anti-inflammatory effects, with special focus on molecular targets which may be significant in safeguarding neurons against AD.

Polyphenols constitute plant-derived compounds that include several phenol structural groups. They help plants defend themselves against pathogen assaults and stress caused by both physical and chemical harm. These compounds provide protection in animals by altering multiple intracellular mechanisms that take care of neurons [19]. Polyphenols are a variety of chemical compounds produced naturally by secondary metabolic processes of plants’, recognized by a number of aromatic rings having a number of hydroxyl compounds. Flavonoids, the predominant phenolic compounds in nature, are increasingly acknowledged for their many benefits. Flavonoid ingestion has been linked to enhancing cognition, reducing neurological inflammation, as well as decreasing oxidative stress in many studies. [20]. Polyphenols have been discovered that they exert their impact on stem cells via a different way. Neurological protective effects by polyphenols and promotion of neurogenesis are seen at several phases, encompassing the regulation of antiapoptotic proteins, activation of intracellular signaling pathways, suppression of oxidative enzymes, and modification related to the function of mitochondria [21]. Furthermore, polyphenols have a significant role in the suppression of free radical species and the binding of metal ions, hence regulating the primary protein degradation processes [22,23].

## 2 Alzheimer's disease

The two primary pathology characteristics of AD is characterized by the presence of senile plaques (SPs) as well as neurofibrillary tangles (NFTs). Till date, multiple experimental results have supported the concept that oxidative stress is linked to an early onset of Alzheimer's disease [24]. The brains of people with Alzheimer's disease have characteristics marked the condition is characterized by a formation of Amyloid-β (Aβ) peptides, referred to as amyloid plaques, with the degeneration of neurons due to neurofibrillary tangles (NFTs), predominantly made up of elevated Tau proteins [25]. The generation of toxic oligomers is essential for the neurotoxic effects of α-synuclein, especially when coupled to Aβ as well as tau.

### 2.1 Aβ aggregation and toxicity

Brain of patients having Alzheimer's disease are characterized by extracellular deposits of Aβ aggregates, known as neuritic plaques [26]. Aβ accumulation and formation begin at the cellular level prior to clinical diagnosis of A.D. Furthermore, new research shows that inflammatory responses may play a substantial role in the development of Alzheimer's disease. Deposition of beta-amyloid (Aβ) polymers induces oxidative stress as well as inflammatory reactions via activation of signaling pathways involving neuronal membranes. The Aβ peptide is a core component of amyloid plaques and is generated from the processing of its parent protein, the amyloid-β protein precursor. Further, Aβ plaques may disrupt the neurotransmitter acetylcholine (ACh), affecting synaptic transmission and stimulating inflammatory pathways which generate reactive oxygen species (ROS) [27]. Plaques of Aβ originate in the orbitofrontal, temporal, and basal neocortex before metastasizing to the hippocampus, amygdala, diencephalon, and basal ganglia. In extreme cases, Aβ might be found in the mesencephalon, lower brain stem, or cerebellar cortex. The buildup of Aβ leads to the formation of τ-tangles in the locus coeruleus, trans entorhinal, and entorhinal areas of the brain. During a critical phase, it reaches the hippocampus and neocortex [28].

In the brain, the proteolytic enzymes cleave the APP to form the Aβ peptide, which has 39-43 amino acid sequences. Amyloid precursor protein (APP), an essential protein on the plasma membrane, is first alteredly cleaved by β-secretases (BACE1) and γ-secretases to form insoluble Aβ fibrils, which is the beginning of amyloid pathology. Aβ then oligomerizes, diffuses into synaptic clefts, and interferes with synaptic signaling [29]. The location where α-secretase cleaves. is beneath the amyloid beta sequence in amyloid precursor protein (APP), which prevents Aβ production. The "amyloid genic pathway" processes APP he processes begins with cleavage by β-secretase, also referred to as β-site APP Cleaving Enzyme 1 (BACE-1), followed by cleavage by γ-secretase, leading to the formation of monomeric Aβ components. Foremost common species are Aβ1-40 and Aβ1-42 [30]. Aβ may be cleared by transporting it into cerebrospinal fluid, crossing the blood-brain barrier (BBB), and being removed by brain macrophages. Besides Aβ plaques and more than 50 % Alzheimer's patients have α-synuclein disease. α-synuclein is a 140 amino acids long protein found in neuronal presynaptic terminals. Research indicates that α-synuclein might serve a function in the progression of Alzheimer's disease beginning with the production of Aβ pathology [31]. Unknown factors may contribute to the disrupted APP metabolism as well as Aβ deposition observed in sporadic instances of AD. However, potential contributors involve intracellular ion equilibrium disruptions, age-related increases in oxidative stress, as well as disrupted energy utilization. Diverse cell types are targeted by the toxicity of Aβ1-42 aggregates, which significantly impacts an extensive array of cellular functions. Disruption in ionic homeostasis, oxidative stress, dysfunction of the mitochondria, stimulation of astrocytes and microglia, and transmembrane disruption are a few of the cellular processes that occur subsequent to the assembly and accumulation of Aβ1-42 [32]. All of these events combine into neuronal network disruption, synaptic disorder, neuroinflammation, and neurodegeneration, that eventually result in the serious dementia that is characteristic of the progressed phases of Alzheimer's disease. Consequently, the disruption of ionic balance induced by Aβ in astroglial cells might have an essential role in their pathological transformation. Significantly, while it is believed that neuro-glial vascular impairment and after that cerebrovascular destruction occur prior to and might even lead to the disruption of Aβ balance, dissolve Aβ clusters that spread across the brain have the potential to accumulate in vascular-level deposits that are insoluble. This can compromise both the functioning of vessels and the functioning of the blood-brain barrier (BBB) [33]. These phytochemicals have the ability to provide therapeutic benefits by acting as antioxidants and reducing inflammation via the regulation of Aβ toxicity. Aggregated Aβ peptides, hydroxyl radicals created by H2O2, and mitochondrial dysfunction caused by APP in Alzheimer's disease may be mitigated by pharmaceutical methods including phytochemicals which maintain mitochondrial dynamics. [34].

### 2.2 Hyperphosphorylation of Tau and its toxicity

Tau hyperphosphorylation is the result of a disproportion in the kinase and phosphatase activities in AD. Aβ aggregates have been identified as potential carriers of Excessive phosphorylation of tau by augmenting the activity of multiple kinases, such as GSK-3β and MAPKs. Furthermore, the stimulation of caspase-3 and calpain-1 is induced by Aβ, resulting in the production of small fragments capable of inducing neuronal mortality and neurite degeneration at the C-terminus of Tau. It is noteworthy that the stimulation of the JNK pathway additionally promotes Tau cleavage by activating caspases. [35]. A difference in kinase and phosphatase activity leads to Tau excessive phosphorylation in Alzheimer's disease. Aβ aggregation may cause Tau hyperphosphorylation by increasing the activity of multiple kinases such as GSK-3β and MAPKs. Additionally, Aβ induces the production of caspase-3 and calpain-1, leading to the cleavage of Tau at the C-terminus, producing tiny fragments that may cause neurite degradation as well as neuronal death. The stimulation of the JNK pathway triggers caspase activity, which in turn promotes Tau breakdown. [36].

**Fig. 1:**
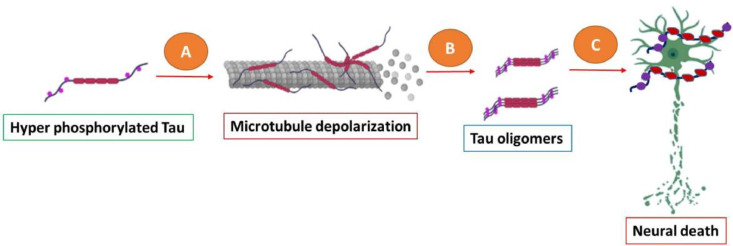
Hyper phosphorylated tau. (A) The detachment of microtubules is induced by tau protein excessive phosphorylation. (B) The accumulation as well as generation of Tau oligomers, that are formed in order to produce tangled neurofibrillary cells. (C) Release of tau oligomers into the outer layer of cells and subsequent neuronal demise.

The role of Tau protein in memory loss is excessively phosphorylated, causing damage to microtubules and interfering with many cellular functions as growth, differentiation, protein transport, including cell structure (Fig. 1). Despite the fact that tau toxicity is recognized as an essential component for neurodegenerative cascade in Alzheimer's disease, the molecular processes enabling tau-mediated neuroinflammation remain poorly understood. A second factor contributing to cell mortality in Alzheimer's disease is the hyperphosphorylation of the tau protein, which is responsible for microtubule stabilization. A high degree of phosphorylation on tau turns it dysfunctional; consequently, the microtubule disintegrates, and the neurotransmitters as well as neuronal signals are blocked by the NFTs.

Tau, an exceptionally soluble protein, is prevalent in neurons of the central nervous system. Its excessive phosphorylation is the result of a kinase-phosphatase imbalance. NFTs are generated when tau hyperphosphorylation transforms the protein from a monomer to an oligomer and then into pair helical filaments (PHFs). In the AD model, the tau oligomer is regarded as the most toxic form that induces synaptic impairment [37]. The brain of AD patients contains three distinct groups of tau: AD tau, which is soluble and hyperphosphorylated; AD Phosphorylated tau (AD P-tau), which resembles normal tau and isn't hyperphosphorylated; and associated helical filaments (PHFs)-tau, which is both insoluble and hyperphosphorylated. Tau levels in Alzheimer's disease brains are approximately 60 % lower than those in healthy brains. The biochemical stability of hyperphosphorylated tau facilitates its aggregation, giving rise to its prion-like characteristics. These findings align with our own investigations utilizing Alzheimer's disease-associated increased phosphorylation tau obtained from a study by Alonso et al. in 1996. The structural transmission from AD P-tau to natural tau resembles the behavior of a prion protein.

Tau phosphorylation has become widely researched since it was discovered that abnormally hyperphosphorylated tau is primary component of PHFs in Alzheimer's disease. 2–3 moles for every mole of phosphate of tau is contained by healthy brain. To date, more than 40 phosphorylation sites have been identified in tau protein isolated from AD brain [38]. Tau has major role is to promote polymerization of micro tubules and stability. Microtubules, a component of the cytoskeletal in all eukaryotes, are made up of heterodimers of α- and β-tubulin creating tubular polymers. Microtubules play a vital role in cytoskeletal integrity and serve as freeways for the intracellular movement of organelles, vesicles, protein molecules, and signaling compounds. [39].

### 2.3 Metal-dependent toxicity of Aβ and tau aggregates and neuroinflammation

**Fig. 2:**
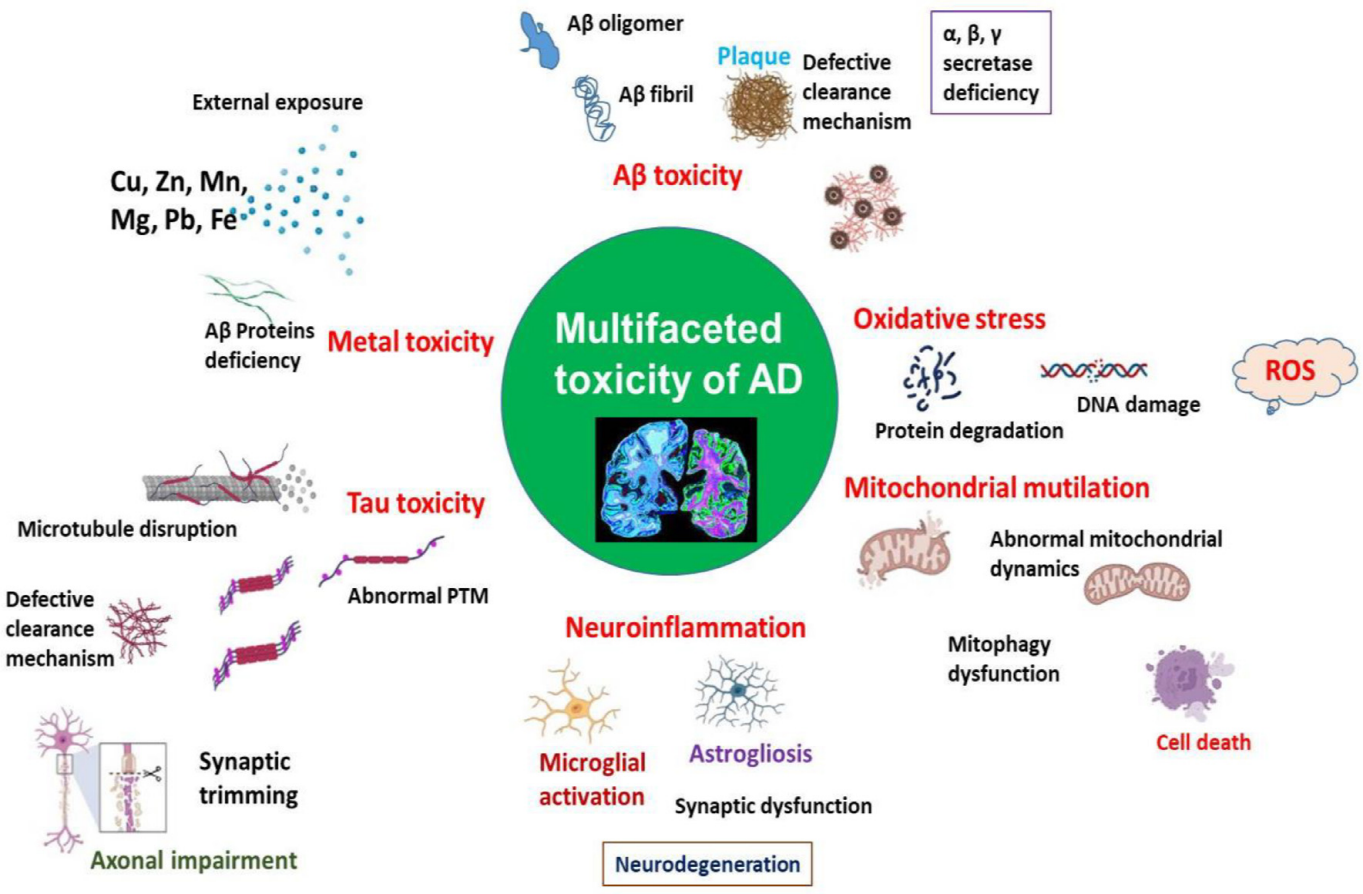
The toxicity of Aβ and tau aggregates in Alzheimer's disease is influenced by metal ions and exhibits a complex and multidimensional nature.

Alzheimer's disease (AD) is a neurodegenerative condition that causes a significant deterioration in cognitive abilities and is accompanied by pathological changes in the brain. The development of AD has been linked to a range of biological and environmental variables, with age being one of the key risk factors. There is a growing body of data indicating that the development of Alzheimer's disease (AD), particularly the accumulation of amyloid and tau proteins, is regulated by the presence of metal ions [40]. Biometal ions, despite their physiological significance, can serve as detrimental cofactors in the etiology of Alzheimer's disease (AD) when their equilibrium is disrupted. Moreover, the widespread presence of environmentally hazardous metal ions and their capacity for rapid dissemination render them a significant worldwide health concern. Although significant advancements have been made in elucidating the impact of metal ions on the aggregation of amyloid and tau proteins, the precise processes and mechanisms involved in this process remain very intricate [41]. Individuals with Alzheimer's disease (AD) have impaired metal homeostasis in their brains, resulting in increased levels of metals that include copper (Cu), zinc (Zn), iron (Fe), and aluminum (Al). (Fig. 2). These metals have a great affinity for the Aβ peptide, causing an increase in toxicity. These metallic ions have been seen to facilitate the process of aggregation and the subsequent production of harmful species. The formation of the Aβ metal ion complex leads to the occurrence of oxidative stress and membrane impairment. Specifically, the Aβ-copper complexes intensify the generation of reactive oxygen species (ROS) and provoke biomolecular damage [40]. The neurons inside the brain exhibit a high degree of susceptibility towards free radicals. The occurrence of DNA damage, protein oxidation, lipid peroxidation, and the formation of advanced glycosylation end products (AGEs) in the brains of individuals with Alzheimer's disease (AD) is commonly associated with oxidative stress caused by free radical assaults and disturbances in metal homeostasis. Oxidative stress has been extensively observed in individuals with Alzheimer's disease (AD) as well as in animal models. dysfunction, of Metal ion such as iron, calcium, the metal copper, and zinc, can contribute to oxidative stress. This oxidative stress, in turn, can lead to tau hyperphosphorylation, Aβ deposition, the formation of cross-links between nerve fibers, and damage to nerve cells. These processes are strongly associated with the development and progression of AD [42].

### 2.4 Oxidative stress and mitochondrial dysfunction in AD

**Fig. 3:**
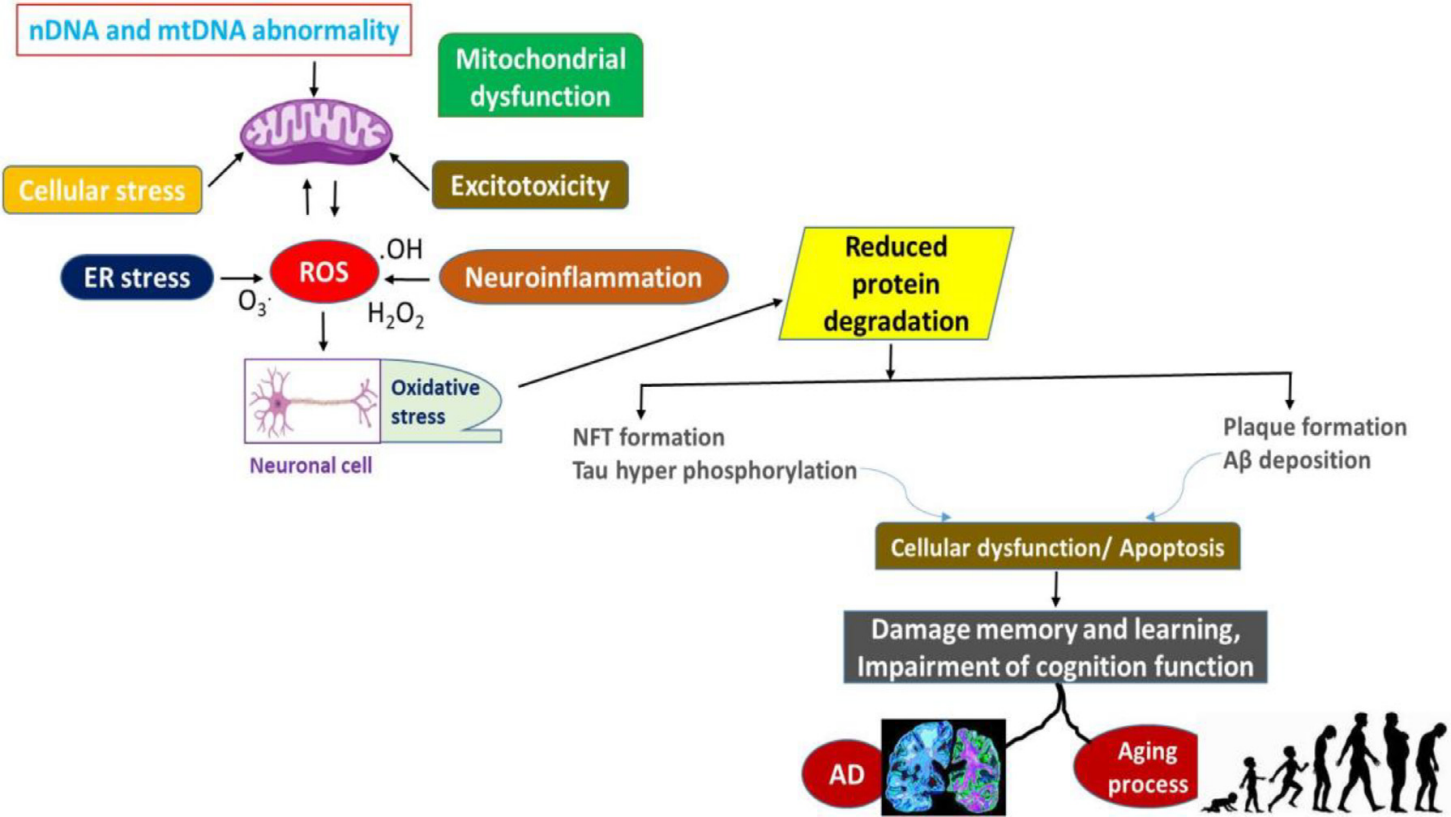
AD develops due to mitochondrial malfunction and oxidative stress in neurons. ROS are often generated by several pathways including ER stress, mitochondrial failure, neuroinflammation, as well as excitotoxicity. High levels of reactive oxygen species (ROS) cause oxidative stress (OS), resulting in mitochondrial malfunction. Oxidative stress inhibits the breakdown of protein molecules and hinders the removal of misfolded proteins, resulting in an accumulation of proteins that results in death of neurons and Alzheimer's disease.

Oxidative stress as well as impairment of the mitochondria have been markers of numerous neurodegenerative disorders, involving AD. An increase in intracellular reactive oxygen species (ROS) levels prompts the peroxidation of lipids, breakdown of proteins, as well as damage to DNA have all been induced in cells of neurons. ROS/microbes target mitochondria extensively.RNS injuries [43]. Furthermore, it is worth noting that ROS and RNS not only involve peroxidation of lipids, protein the carbonylation process, along with mitochondrial destruction of DNA. but they also initiate the development of the permeable transition pore (PTP), a process that enhances the signals generated by free radicals. ROS (comprising H2O2, OH, and O2) might be the underlying cause of developmental abnormalities in the brain of humans and defects in mitochondrial respiration that are accompanied by increased ROS production. Furthermore, they play a role in the dynamic modifications that occur in the brain as AD and aging advance (Fig. 3). The relationship between oxidative stress (OS) and AD has been extensively acknowledged as a prodromal factor [44]. Cellular regulation of OS is crucial for preserving the equilibrium of the microenvironment, which is required for numerous biological processes including bioenergetics, vesicle transport, and post-transcriptional modifications. Due to their membranes being dense with fatty acids that are polyunsaturated and their typically low antioxidant level, neurons are especially susceptible to OS [45]. Age-related oxidative damage significantly impairs synaptic components that are implicated in neuronal plasticity, cytoskeletal dynamics, and cellular communication etc which are recognized as defective mechanisms in Alzheimer's disease. A reduction in the concentration of presynaptic high-affinity choline transporter 1 (CHT1) was detected in synaptosomes located in the hippocampus as well as neonatal cortex of human with Alzheimer's disease. In addition, elevated OS deactivates nAChRs, which results in cholinergic transmission being inhibited permanently [46]. Additionally, ROS can modulate the function of the BBB by upregulating the expression of a number of metalloproteinases, specifically isoform 9. MMP9 expression is significant in the microvascular environment of the brain because its modifications are linked to an increase in BBB permeability, which promotes the progress of AD by allowing inflammatory factors and reactive oxygen species to extravasate into the brain [47]. There is an abundance of polyunsaturated fatty acids in brain tissue. They are susceptible to degradation into malondialdehyde, a compound that induces cellular harmful stress and destruction of DNA [48].

The brain is very susceptible to oxidative stress due to its high concentrations of redox transition metal ions, rapid oxygen consumption, and higher quantities of polyunsaturated fatty acids (which are easily targeted by free radicals). Additionally, the brain contains an exceptionally low concentration of antioxidants. [49]. Indeed, the accumulation of Ab protein induced by reactive oxygen species (ROS) in Alzheimer's disease leads to neuronal mortality by causing lysosome membrane breakdown. The most prevalent mitochondrial electron transport chain (ETC) malfunction in Alzheimer's disease (AD) involves a cytochrome c oxidase deficit which causes an increase in reactive oxygen species (ROS) manufacturing, a reduction of stored energy, and a disturbance in the utilization of energy [50]. In addition, reactive oxygen species (ROS) prevent phosphatase 2A (PP2A), thereby promoting the stimulation of glycogen synthase kinase (GSK) 3b, which is among the protein kinases implicated in tau phosphorylation.

**Fig. 4:**
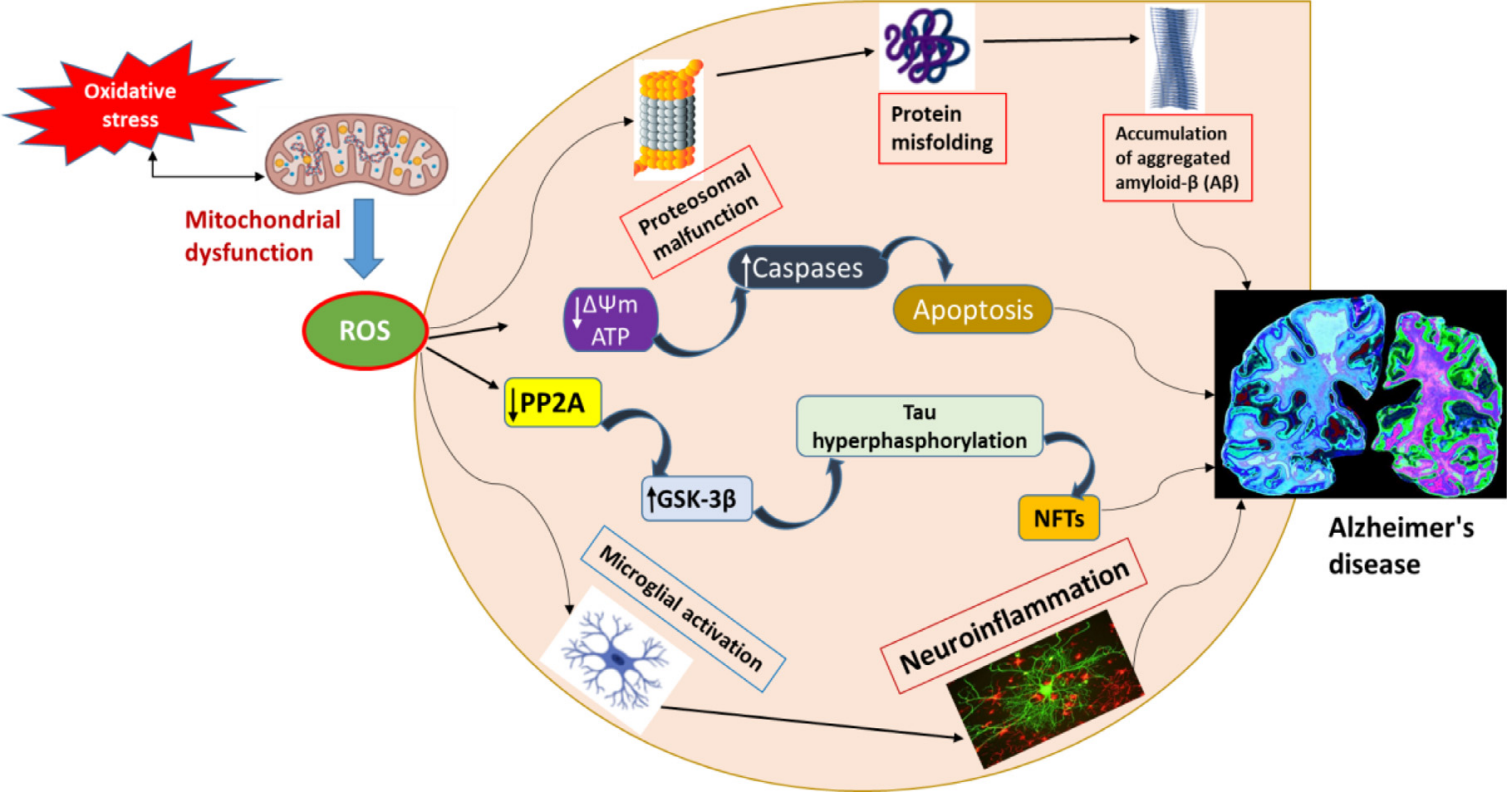
Depiction of ROS-triggered mitochondrial irregularities in Alzheimer's disease.

AD has been associated with oxidative dysregulation and a significant increase in its byproducts, according to multiple reports. Extensive research has established that Alzheimer's disease significantly amplifies lipid peroxidation, which occurs when reactive oxygen species (ROS) employ a free radical chain reaction mechanism to attack lipids and generate lipid peroxidation products [51]. Progressive mitochondrial dysfunction has also been implicated in aging and Alzheimer's disease as the primary source of reactive oxygen species (ROS) production; mitochondria are a main target of oxidative damage. As an essential factor in the pathogenesis of Alzheimer's disease, mitochondrial dysfunction resulting from the improper processing of reactive oxygen species has been documented in numerous studies [52]. Furthermore, it is critical to mention that oxidative stress is associated with mitochondrial functioning, not only due to the fact that mitochondria produce reactive oxygen species (ROS), but also for the reason that ROS can induce a decline in mitochondrial function (Fig. 4). Reducing ROS levels through the use of antioxidant pharmaceuticals, exercise, and dietary modifications may therefore protect neuronal mitochondria from oxidative injury and thereby reduce the risk of AD.

When there is either excessive ROS synthesis or a dysfunctional antioxidant system, it leads to an imbalance in cellular redox status, resulting in an overproduction of ROS. Reactive oxygen species produced during the process of cellular respiration harm mitochondria and impair neural function. Elevated levels of reactive oxygen species (ROS) lead to a decrease in mitochondrial membrane potential (ΔΨm) and adenosine triphosphate (ATP) production by impairing mitochondrial energy reserves, disrupting energy metabolism, and compromising mitochondrial dynamics and mitophagy. ROS also leads to elevated caspase activity and triggers apoptosis. Conversely, excessive ROS generation leads to the suppression of phosphatase 2A (PP2A), resulting in the activation of glycogen synthase kinase (GSK) 3β, leading to tau hyperphosphorylation as well as the buildup of neurofibrillary tangles.

Mitochondria are obligatory organelles in all mammalian cells because they regulate apoptotic pathways as well as energy metabolism, both of which are critical for cell survival or demise. However, their function extends beyond that, as neurons require significant amounts of energy, specifically for synaptic activity within neurons and for neurotransmission events. [53]. Under conditions of elevated OS, the serotoninergic efficiency of mitochondria is compromised due to membrane permeability and modifications in serotoninergic metabolism. Overall, mitochondrial dysfunction diminishes monoaminergic activity. Different in vitro studies have postulated a correlation between increased levels of Aβ, dysfunctional mitochondrial function, and oxidative burden, all of which aid in the development of Alzheimer's disease. In Alzheimer's disease, a reduction in neuronal ATP levels signifies mitochondrial dysfunction, which is correlated with an excessive generation of reactive oxygen species (ROS) and suggests mitochondria might be unable to sustain cellular energy. A considerable quantity of ATP is depleted within the brain as a result of high energy demands of neurons as well as glia. Significantly, dysfunction of mitochondria is contributing to decreased ATP production, Ca2+ dyshomeostasis, and ROS production. Early-stage Alzheimer's disease is characterized by mitophagy and mitochondrial metabolism alterations, but its root causes remain inadequately understood. Therefore, research that uncovers the mechanisms underlying mitochondrial dysfunction in Alzheimer's disease will contribute to the development of therapeutic approaches that safeguard synaptic activity and, consequently, cognitive function, thereby enhancing our knowledge of the disease progression of neurodegenerative disorder. Ca2+ is an essential regulator of important neuronal processes, including production, motility, metabolism, plasticity of synapses, proliferation, expression of genes, and necrosis. Mitochondria are crucial in maintaining cellular Ca2+ homeostasis. It is widely accepted that dysregulation of Ca2+ homeostasis is a crucial factor in the acceleration of pathology associated with AD. By stimulating the permeability transition (PT), mitochondrial Ca2+ can easily transform into a toxic factor. Intracellular Ca2+ maintains numerous neuronal functions; however, a disruption in its homeostasis may result in neuronal damage or demise. In fact, elevated cytosolic Ca2+ concentrations may induce the most significant damage processes, namely mitochondrial Ca^2+^ accumulation and subsequent dysfunction [54]. However, an abundance of results from experiments indicates that mitochondrial dysfunction as well as oxidative stress both contribute to an upregulation of amyloidogenic cleavage of the APP and an abnormal production of Aβ. In fact, research has demonstrated that oxidizing compounds can enhance the activity and expression of BACE-1 and the APP [55]. Furthermore, the Aβ peptide has been identified as a target molecule for metal-catalyzed oxidation in both in vitro and in vivo investigations [56]. Nevertheless, the precise mechanism by which Aβ oxidation affects its aggregation and/or its affinity for plasma membranes is still unknown.

## 3 Pathogenic mechanism in Alzheimer's disease

Alzheimer's disease (AD) is a complex neurodegenerative disorder that occurs with the passage of time and has numerous etiological factors. Extracellular amyloid -peptide (Aβ) plaques along with intraneuronal neurofibrillary tangles (NFTs) composed of tau protein that has been hyperphosphorylated constitute the most prominent characteristic of this disorder. Neuronal synapses are subsequently lost, and neuronal degeneration occurs. As a consequence, critical neurotransmitters experience a reduction in the amount [57]. The primary constituent of amyloid plaques, Aβ, is obtained through proteolytic cleavage of APP. The hypothesis that APP and Aβ play a crucial role in the development and progression of AD [58]. NFTs are composed of forms of the microtubule-associated protein tau that have been hyperphosphorylated. Tau protein association with microtubules is accountable for cytoskeleton stability in neurons. Due to its limited binding for microtubules, hyperphosphorylated tau remains insoluble, putting microtubule regulation at risk and consequently leading to synaptic dysfunction and neurodegeneration. Inflammation, oxidative damage, tau kinase increase, and phosphatase reduction all contribute to tau hyperphosphorylation [59]. Neurovascular endothelial cells are adversely affected by Aβ, which can occur through direct action or by inducing local inflammation. The production of Aβ in the cerebral microvasculature is induced by inflammation, and this Aβ subsequently promotes the secretion of pro-inflammatory mediators [60]. At the beginning, Alzheimer's disease (AD) was perceived as a disorder associated with an increase in the production of Aβ and a decrease in its degradation. Concurrently, inadequate approval is identified as a co-factor. Brains affected by Alzheimer's disease (AD) contain Aβ plaques that are intimately related to hyperactive microglial cells as well as reactive astrocytes. These cells display increased levels of cytokine and acute-phase protein expression. Microglia, which are mononuclear phagocytes, are found in the brain. Their primary function is to maintain brain homeostasis and prevent harmful damage to the central nervous system. Microglial cells that sustain a neuroprotective role in healthy individuals through the clearance of Aβ plaques [61]. Additionally, they exhibit the expression of a variety of neurotrophic factors, including nerve development factor, brain-derived neurotrophic factor (IGF)-1, and transformation growth factor-β. In conditions of systematic or regional inflammation, cellular and molecular constituents transmit inflammatory signals to the brain via distinct pathways. In typical circumstances, the inflammatory response is appropriately regulated in order to prevent uncontrolled injury caused by inflammation [62]. Thus, rather than addressing these systemic inflammatory signals with a protective response, the damaged microglia evoke an exaggerated reaction that contributes to the etiology of AD. The microglia that have been "energized" alter in appearance and secrete numerous cell antigens. Such cells are called "activated microglia." When microglia are activated, an array of pro-inflammatory substances are secreted. In an experimental mouse model of Alzheimer's disease, tau hyperphosphorylation is caused by chronic inflammation and cytokine activation [63].

**Fig. 5:**
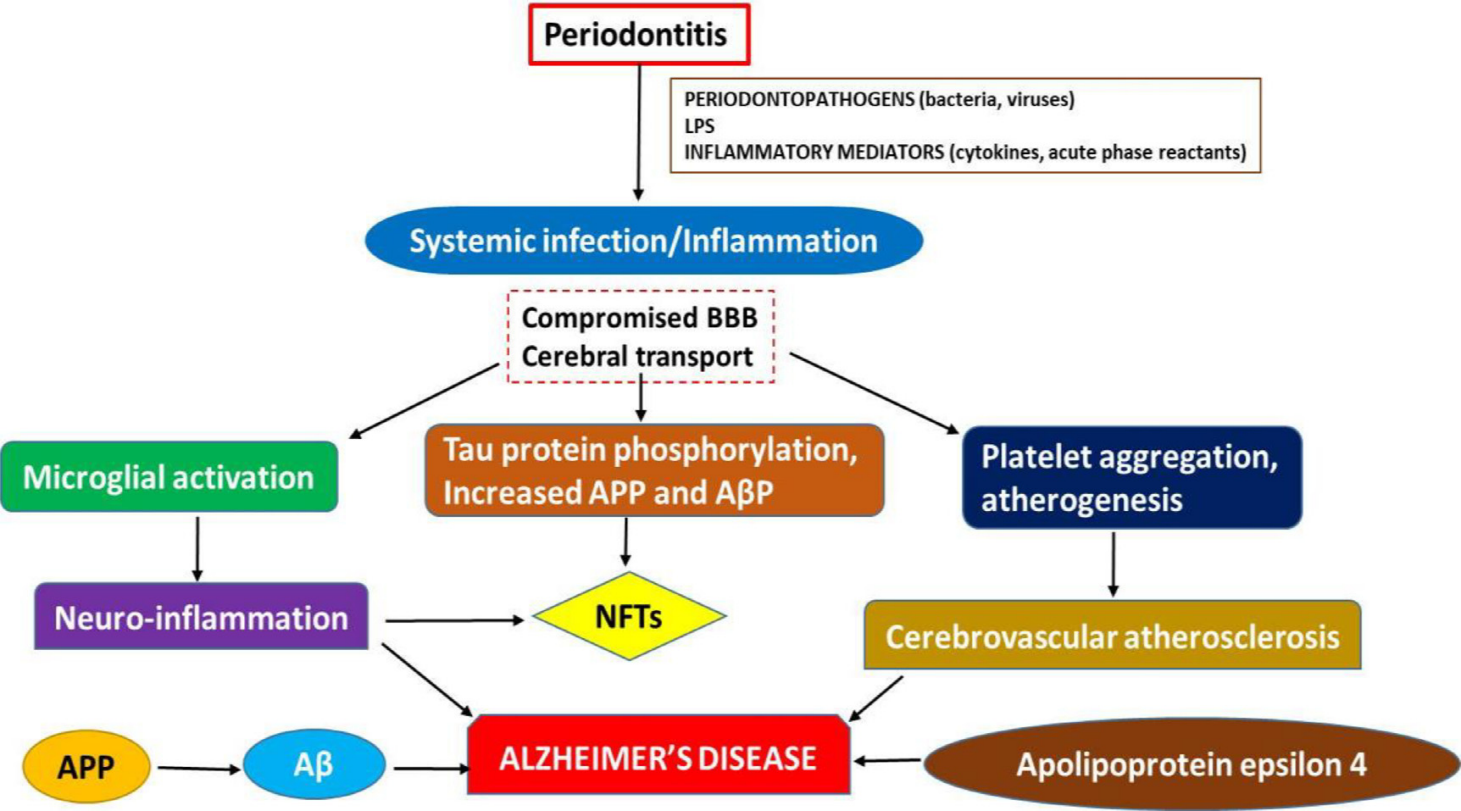
Potential mechanisms underlying the development of Alzheimer's disease. **LPS**-lipopolysaccharide; **BBB**-blood brain barrier; **APP**-amyloid precursor protein; Aβ-amyloid beta; NFTs-neuroofibrillary tangles.

Concurrently with periodontal inflammations and atherosclerotic cardiovascular diseases (ACD), inflammatory systemic markers such as interleukins, TNF-, and reactants in the acute phase boost in concentration. There are two plausible mechanisms by which periodontitis may contribute to the advancement of Alzheimer's (Fig. 5):

• Periodontitis preceding systemic inflammation/infection
• Bacterial and viral influence

Pro-inflammatory amounts of cytokines are increased by pathogenic periodontal bacteria as well as the host response, according to the first process. A wide range of pro-inflammatory substances and cytokines are secreted into the circulatory system, thereby contributing to the overall inflammatory stress in the body. Pro-inflammatory compounds have the ability to breach the blood-brain barrier (BBB) as well as infiltrate the cerebral cortex. The second process may include the infiltration of the brain by bacteria and viruses present in the tooth plaque biofilm. This phenomenon could take place either peripherally or directly by means of cerebral transport facilitated by the circulation [64]. A substantial body of research implicates inflammatory pathways in the central nervous system as the causative agents of memory loss observed in Alzheimer's disease. Research conducted on mouse models has demonstrated the beneficial impacts of anti-inflammatory agents in reducing the accumulation of amyloid plaque and neuroinflammation. The study conducted by Yan et al. [65] identified notable reductions in glial fibrillary acidic protein and IL-1β levels, in addition to a substantial eclipse in plaque burden, in rodents that were administered non-steroidal anti-inflammatory agents. The periodontal plaque spirochetal species exhibit a diverse array of virulence factors that facilitate their engagement with the defenses of the host and enhance their capacity to infiltrate the periodontal tissues in the host. [66]. Peri-periodontitis is characterized by persistent periodontal inflammation, which provides an ongoing supply of systemic pro-inflammatory factors that are elevated. These agents and their byproducts possess the ability to penetrate the blood-brain barrier (BBB) and gain access to the brain. Negative effects may be caused by these compounds either through direct or indirect impact on the brain through the disruption of vascular integrity [67]. Spiketal species that invade the brain are capable of sustaining an ongoing persistent inflammatory process through the stimulation of innate immune responses, which involve the involvement of diverse signaling pathways. This activation ultimately leads to neuronal degeneration. There is a proposition that inflammation could serve as a mysterious mediator connecting periodontitis and the development of Alzheimer's disease. "Undesirable systemic inflammation" can be exacerbated by periodontitis, which can also increase the systemic the biological burden. It could potentially be considered one of the risk factors contributing to the progression of neurodegeneration in Alzheimer's disease.

## 4 Multi-target therapies and lifestyle intervention strategies in Alzheimer's disease

The intricate characteristics of neurological disorders have generated an urgent requirement for the development of multitarget-directed ligands capable of targeting the complementary processes implicated in such conditions. In the development of therapeutics for AD both cholinesterase as well as β-secretase enzymes serve as the primary targets. A potential multitarget agent was identified in 2018 in the form of a novel pyrimidinylthiourea a byproduct modified with propargylamine. This compound exhibited a strong affinity for inhibiting AChE and MAO-B in the nervous system of mice, and it also mitigated the cognitive impairment induced by scopolamine in Alzheimer's disease in mice [68]. Iron metabolism alterations are a frequent complication of neurological disorders. There exists a correlation between the buildup of iron within the brain of an individual and an excessive production of reactive oxygen species. This surplus ultimately leads to neuronal degeneration and a deterioration in mental abilities. Additionally, prior studies have demonstrated that iron is essential for inhibiting the development of amyloid-β aggregates which are organized, an event that contributes to neurodegeneration in Alzheimer's disease. [69]. Therefore, the maintenance of cerebral iron homeostasis is recognized as a promising therapeutic target for conditions associated with aging. M30, a multitarget neuroprotective material, was synthesized to exhibit dual iron eliminating capabilities as well as inhibitory effects on MAO-A as well as -B. Multitarget directed ligands (MTDLs) are efficacious agents for addressing the incomprehensible intricacy of neurological disorders. The capacity of MTDLs to target different pathological cascades of neurodegenerative diseases is equivalent to their enhanced therapeutic properties in comparison to single-target tiny compounds. Consideration has been given to the multi-target approach to AD developing medicines in recent years, but it has not yet acquired widespread acceptance or been implemented by the scientific community. This may be due to the fact that the single-target approach is the conventional, standard method for developing traditional drugs, which is also more readily accepted by the FDA approval process. One possible explanation could be the difficulties associated with formulating this approach and the intricacy of assessing the mechanisms underlying the drug's effects. Develop an initial pharmaceutical compound comprising at least two functionally active groups or targets. Multiple molecular pathways can be targeted by numerous natural and synthetic compounds that contain multiple functional groups. In the realm of conventional pharmaceutical development, non-specific activities encompass anything beyond the intended target activity. Frequently, the greatest potential specificity is desired in a drug choice. If "non-specific actions" target other mechanisms implicated in AD, however, less specific drugs may be favored over more specific ones when it comes to the treatment of multifaceted AD. Apart from vaccines as well as enzyme inhibitors, various compounds have been developed to exert a wide range of techniques for action. ALZT-OP1, a Phase III clinical trial involving a mixture of two pharmaceuticals that effectively prevents Aβ aggregation and neuroinflammation, is presently taking place [70]. Decades have been devoted to the investigation of neurotrophins (NTs) and receptor-based treatments for Alzheimer's disease (AD) due to pleiotropic impacts of NTs and receptor signaling. However, the practical implementation of NTs is constrained by their inadequate pharmacological characteristics, including brief plasma half-lives, inadequate oral bioavailability and BBB permeability, and restricted diffusion into brain tissue [71]. The transmission of genes technique has been employed as an alternative approach to develop therapies for AD based on NTs. In addition, evaluate organic compounds with numerous processes of action against the diverse toxins and mechanisms implicated in AD. A number of naturally occurring compounds, including derivatives of resveratrol, chelerythrine, chalcone, coumarin, huprine, curcumin, rhein, and berberine, have demonstrated progress in search of developing drugs for AD [72]. The progression of Alzheimer's disease (AD) and different molecular pathways are both susceptible to modulation by specific molecules or elements. An illustration of this is the regulation of neurodevelopment, neural plasticity, neuronal survival, and neurodegeneration through brain insulin signaling [73]. Neurodegeneration in Alzheimer's disease (AD) is likely associated with disrupted brain glucose metabolism, a well-documented metabolic abnormality that precedes the disease and appears to be influenced by dysfunctional insulin signaling in the AD brain. The research of oral medications that enhance insulin resistance or sublingual treatment with insulin to restore brain insulin signaling for the management of Alzheimer's disease (AD) is now being implemented [74]. The diversity of AD patients' molecular mechanisms, pathologies, and clinical symptoms is predicated on a multifaceted character of the disease. However, active Aβ immunotherapy as a potential preventive measure contrary to AD has yet to undergo rigorous testing, and numerous safety concerns, including the intensity of immune reactions for the vaccine, require additional investigation.

It appears that lifestyle interventions modulate a variety of molecular mechanisms to minimize morbidity and mortality in aging populations; thus, they may represent effective non-therapeutic approaches to address metabolic and aging-related diseases. Innovative approaches that target a range of cardiovascular and metabolic risk factors-such as a lack of exercise cigarette smoking, postmenopausal high blood pressure, midlife being overweight, and diabetes—may be able to reduce the incidence and prevalence of decline in cognition and AD, according to recent studies [75]. Consequently, dietary and lifestyle modifications may serve the same effective primary prevention approaches for Alzheimer's disease. Adiponecteritis and Alzheimer's disease (AD) may be influenced by neuroinflammatory processes that are altered by nutritive dietary components that are healthy and abundant in anti-inflammatory and antioxidant qualities. Sultana and Alauddin [76]. The utilization of these nutritional supplements and behavioral patterns could potentially serve as possible strategies to avoid cognitive impairment or postpone the onset of Alzheimer's disease. The functions of various food sources in both good and bad health have been investigated, including omega-3 fatty acids, nutraceuticals, minerals, micronutrients, and vitamins. In the pathophysiology of diabetes, obesity, cardiovascular diseases, cancer, and so forth, these nutritional strategies are recognized to ameliorate the condition. Curcumin is extracted from the rhizome of Curcuma longa, produced primarily in India and China, It is recognized for its significant contribution to disease prevention via the regulation of diverse biochemical pathways [77]. It is the primary active constituent of turmeric, a herbaceous Asian spice. Antioxidant, anticancer, anti-inflammatory, antibacterial, antiviral, and antifungal properties distinguish turmeric powder from other substances that are traditionally treated with it. Curcumin exhibits neuroprotective, anti-inflammatory, and potent antioxidant properties, which have been demonstrated in recent research to confer a protective effect against Ab in AD [78].

Flavonoids, which have a polyphenolic construction, are prevalent in a variety of natural sources including fruits, vegetables, cereals, foliage, roots, stems, florals, tea, and wine (Panche and Diwan). Flavonoids are classified into diverse subclasses based on their chemical makeup, including but not limited to flavonols, flavones, anthocyanins, isoflavones, chalcones, and dihydrochalcones. Flavonoids may possess potent antioxidants, antibacterial, anti-mutagenic, and anti-carcinogenic properties, according to a number of studies. As a result of these characteristics, flavonoids prevent the development of cancer, Alzheimer's, and cardiovascular diseases. [79,80].

Quercetin, a flavonoid found in a wide variety of foodstuffs as well as vegetation, includes American elder, red wine, scallions, green tea, pears, cherries, and Ginkgo biloba. Possible upregulation or downregulation of cytokines via the nuclear factors (Nrf2), Paraoxonase-2, c-Jun N-terminal kinase (JNK), Protein kinase C, Mitogen-activated protein kinase (MAPK), and PI3K/Akt paths are among molecular processes beneath neuroprotective benefits of quercetin, as shown in in living cells and in the laboratory research. [81].

Abnormalities in the brain of aged APP rodents are reversed and reduced by caffeine. The observed decrease in Ab plaques could potentially be attributed to the upregulation of survival pathways in the brain and the stimulation of protein kinase A activity, as suggested by Zeitlin et al., [82]. This is supported by the elevated levels of phosphor-CREB and the reduced production of phosphor-JNK as well as phosphor-ERK in animal models of Alzheimer's disease.

Resveratrol, a polyphenol found in red wine and grapes, is gaining recognition as a result of its powerful antioxidant and anti-inflammatory properties. The previously mentioned characteristics of resveratrol are attributable to its molecular form, which permits it the capacity to form bonds with numerous biomolecules. Resveratrol has been identified as an activator of the class III HDAC sirtuin 1 (SIRT1), which protects cells from ROS-induced inflammation and oxidative damage [83]. Supplementing with resveratrol, which possesses potent antioxidant, anti-inflammatory, and neuroprotective attributes, could potentially serve as an effective therapeutic approach in addressing the increasing incidence of cognitive impairment and Alzheimer's disease.

Increased dietary mineral intake protects against a variety of metabolic diseases, including type 2 diabetes, hypertension, stroke, and cognitive decline, according to substantial evidence. In cognitive decline and Alzheimer's disease, an excess of reactive oxygen species (ROS) is linked to malfunction of the mitochondria, as evidenced by changes in biosynthesis and metabolism [84]. Drugs that target mitochondria may represent a viable medicinal approach in the treatment of neurological disorders and aging.

Vitamins may be beneficial in preventing the development of Alzheimer's disease and preserving cognitive function, as they perform essential functions in the nervous system [85]. It has been discovered that vitamin supplements significantly reduce the risk of long-term diseases, such as cancer and cardiovascular disease. In numerous diseases, these dietary changes target molecular mechanisms implicated in disease pathophysiology, such as calcium homeostasis, inflammatory pathways, oxidative stress, and mitochondrial dysfunction. Numerous molecular mechanisms implicated in the pathogenesis of Alzheimer's disease (AD), mild cognitive impairment (MCI), and aging were modulated in a recent study, demonstrating the function of vitamins in these conditions. Russell et al. [86] found that regular exercise improves mitochondrial health in the skeletal muscles by stimulating multiple cell signaling pathways. It is recognized for its ability to regulate blood sugar and body weight, support blood pressure, diminish dyslipidemia, and enhance the health of muscles and bones. By enhancing metabolic health, calorie restriction (CR) is an additional potentially effective non-pharmacologic strategy that halts brain aging. Through the neutralization of the detrimental effects of ROS and oxidative damage, CR is effective. By targeting sirtuins, CR has been demonstrated to prevent the development of numerous diseases.

## 5 Role of phytochemicals in the prevention of Alzheimer's disease

**Fig. 6:**
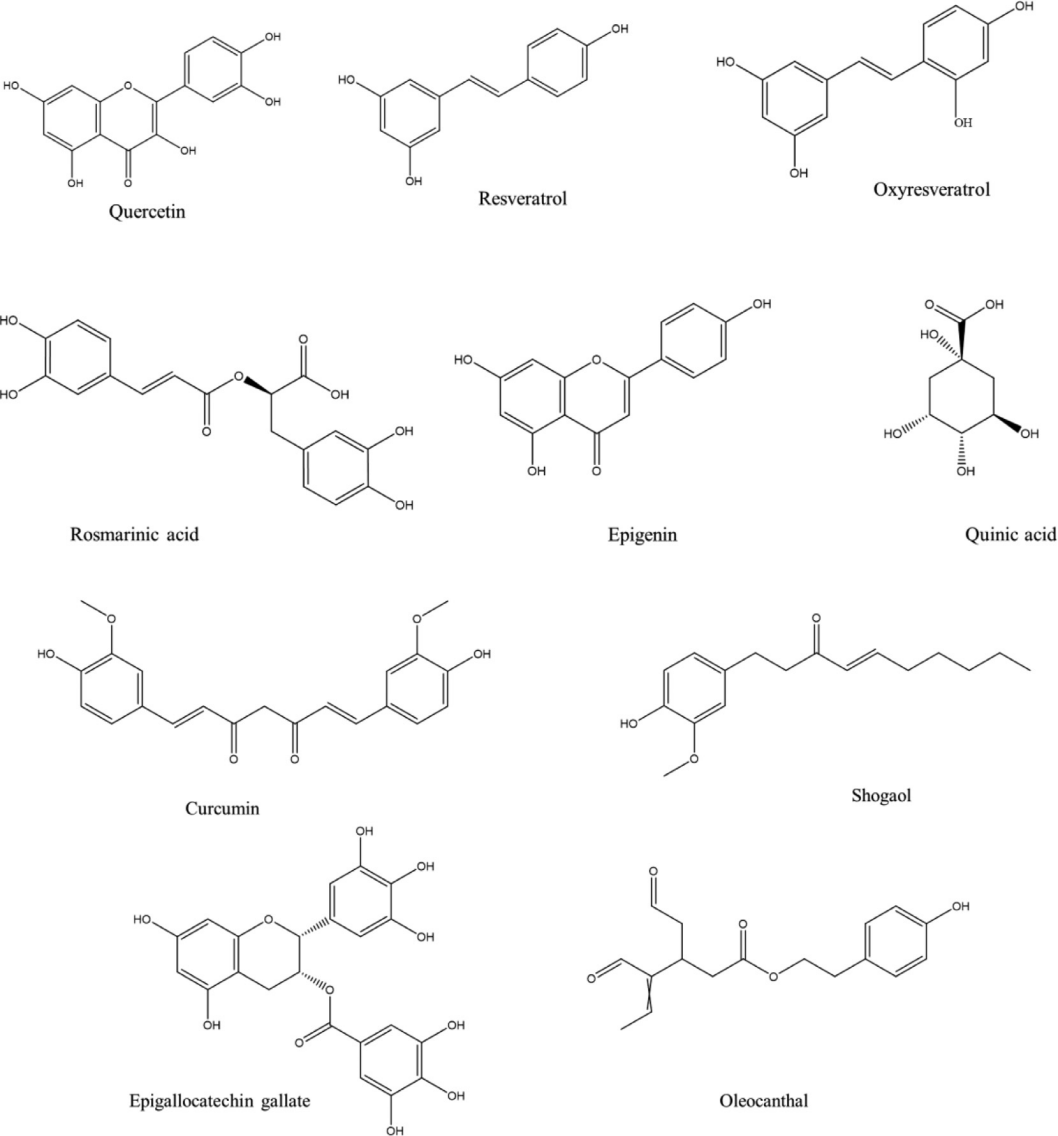
Chemical structure of some phytochemicals.

Phytochemicals, including organosulfur, phenols, terpenes, as well as terpenes, have been derived from plant compounds that impart coloration, smells, and irritating properties. These substances serve as protective barriers against both internal and external stresses, including metabolic processes such as free radical reactive oxygen species (ROS) and expression of proteins as well as hazards from the environment including infectious agents, enemies, and UV rays, which are detrimental to plant survival. Polyphenols (broad assemblages of phenols, which are compounds consisting of an aromatic ring bonded to a hydroxyl group) are produced by numerous plant species. Typical phytochemicals such as stilbenoids, phenolic acids, along with flavonoids are included. It has been demonstrated that a number of phenolic acids regulate neuropathological processes associated with AD. (Fig. 6).

### 5.1 Role of dietary phytochemicals

Curcumin, a phenolic acid, is present in turmeric, a vibrant yellow root spice that is botanically associated with ginger. Morphological stains thioflavin-S and Congo red, that are utilized of illustrating amyloid fibrils in brain tissue, resemble it morphologically. It was stated in experiments that dietary curcumin can inhibit developing deficiencies, synaptic injury, oxidative stress, and cortical microgliosis in rodents following intracerebroventricular injection of Aβ [87]. Heme-Aβ peroxidase harm to muscarinic ACh receptors were also diminished, along with Aβ plaques as well as oxidative stress on APP recombinant rodents.

Furthermore, an investigation involving transgenic mice revealed elevated levels of DNA damage in comparison to control mice. The researchers documented that the damage could be substantially minimized through adding supplements to the diet with curcumin [88]. The gastrointestinal microbiota is also highly implicated in the metabolism and fermentation of dietary phytochemicals, in along with its function in the pathogenesis of AD. Initial absorption of dietary phytochemicals may have shown to range from 5 to 10 %. Phytochemicals that persist are transported to the colon, after which they are subjected to thorough metabolism by microbiota. While the precise pathways of metabolism and molecular targets remain unknown, it is possible that the beneficial properties of dietary polyphenols could be enhanced through the intestinal microbiome's degradation of them [15]. Regular curry consumers are associated with improved cognitive function and a reduced risk of Alzheimer's disease; the compound responsible for these benefits was identified as curcumin, which is present in turmeric, an important component in curry [89]. Curcumin is being documented to safeguard the digestive system and microbial metabolism through analogous mechanisms [90]. These mechanisms include inhibiting oxidative bowel injury, maintaining equilibrium in gut immune reactions, and promoting intestinal epithelial cells. An estimated 90–95 % of the polyphenols found in food are retained in the colon, where they are broken down by the bacteria in the gut into easily absorbed small biological phenol compounds [91]. This is because of the prohibitive molecular weight of the polyphenols in the tiny intestines. Curcumin exhibits limited absorption in the small intestine when administered orally (1 % bioavailability) and fails to penetrate the blood-brain barrier (BBB), a property similar to its insolubility. Reduce pathologies associated with microbial metabolites, which mediate microbe-produced neurotransmitters and and prevent the transfer of microbial organisms and their products are all functions of polyphenols in the brain-gut-microbiota system. [91].

Anthocyanins, including their deglycosylated form anthocyanidins, are prevalent in fruits, vegetables, certain pigmented cereals, and legumes. These water-soluble flavonoids have shown promise as potential interventive agents in Alzheimer's disease. Furthermore, it has been documented that Mexican blue maize anthocyanins inhibit α-amylase, thereby preventing the digestion of starch. Furthermore, the unprocessed starch can serve as a source of energy for gut probiotics (specifically Lactobacilli along with Bifidobacterium) and as precursors for the metabolism of SCFA-producing microorganisms. [92].

Tea has been utilized for health benefits for numerous centuries on account of its potent phenolic content. Among these, epigallocatechin gallate (EGCG), which comprises around 10 % of the dried weight of tea leaf, is one of the most prevalent compounds [93]. It was discovered that EGCG inhibits the development of curli-related proteins, thereby decreasing the amount of amyloid generated by gastrointestinal microbes; this may account for the decreased serum amyloid level. Tea/EGCG intake has been associated with ameliorative impacts on neurodegenerative impairments such as memory loss and cognitive dysfunction, according to a number of clinical trials. As an illustration, epidemiological investigations have provided evidence that consistent consumption of green tea mitigates cognitive decline and dementia associated with aging, with EGCG being identified as the most efficacious compound [94]. Multiple research studies have suggested that the administration of dietary polyphenols, including (−)-epigallocatechin-3-gallate (EGCG) and curcumin, can mitigate age-related cellular damage through the inhibition of reactive oxygen species (ROS) production.

Terpenes comprise an extensive array of lipid-soluble substances that manifest a spectrum of toxicity ranging from lethal to entirely edible. This, in conjunction with their aesthetically pleasing scents and tints, aids in their multifaceted ecological functions, which encompass protection, discouragement, and promotion of pollination [13]. Advanced exchanges with insects are facilitated through direct neural system (CNS)-to-insect relationships via neurotransmitters, hormones, and cholinergic systems; these interactions may be applicable to the mammalian CNS and offer intriguing prospects for the development of therapeutics for AD.

It was discovered, for instance, that ginsenosides safeguard gastrointestinal barrier functions by increasing glucose absorption by intestinal epithelial cells, thereby preventing the translocation of microbial cells and byproducts to the central nervous system. Certain microbes that are upregulated include Bacteroides, Lactobacillus, and Bifidobacterium. These microbes have been identified as ginsenoside deglycosylators, which convert ginsenosides into secondary compounds that enter the systemic circulation more readily and exhibit more potent anti-inflammatory effects than the parent compounds [95]. The dual mechanism of action exhibited by ginsenosides significantly amplifies their neuroprotective properties, rendering them viable candidates for incorporation into preventive and therapeutic strategies targeting AD.

A study conducted by Zhou et al. [96] discovered that xanthoceraside, which was extracted from the husks of Xanthoceras sorbifolia Bunge, increased the level of production of BDNF (brain-derived neurotrophic factor), suppressed tau excessive phosphorylation and accumulation, and mitigated oxidative stress, neuroinflammation, as well as synaptic damage while having no impact on AChE activities. By virtue of its the insoluble nature, xanthoceraside has a difficult time traversing the BBB and entering the circulatory system [97]. Consequently, the brain-gut-microbiota axis determines how its bioactivities regarding AD are manifested in relation to relationships with the gut microorganisms. An improvement in memory among older people was found to be associated with particular dietary phytochemicals, including carotenoids, although no single nutrient stands out. Nevertheless, through meticulous strategizing, research endeavors can be formulated to specifically examine the correlation between age-related cognitive impairments as well as polyphenolic nutritional supplements; the outcomes may prove to be more illuminating. However, in order to ascertain that these studies produce valuable nutritional insights, further preliminary research employing suitable simulators is necessary to validate the beneficial implications that have already been published.

### 5.2 Antioxidant role of phytochemicals in AD

After plants have been put under adverse conditions, an oxidative surge may induce an imbalance among the generation and removal of reactive oxygen species (ROS), which can activate both biochemical and nonenzymatic reactive antioxidant processes. The initial one pertains to alterations in the functionality of enzymes that protect against free radicals, including peroxidases, catalase, and superoxide dismutase. Conversely, the non-enzymatic response concerns the production of antioxidants with both moderate and high molecular weights (ascorbic acid, glutathione, carotenoids, phenolic acids, flavonoids, and others; tannins). Significant research focus is devoted to examining the antioxidant capacity of naturally occurring compounds, also known as phytochemicals.

Carotenoids, which impart a significant number of the red, orange, and yellow colors to fruits, foliage, and flowers, are effective antioxidants. Their antioxidant activity is predicated on their capacity to scavenge peroxyl radicals; they are abundant in fruits and vegetables, where they are easily accessible [98]. The effectiveness of reducing is correlated with the total amount of conjugated double bonds present within these compounds; for instance, ***U***- and *α*-carotene, as well as zeaxanthin as well as cryptoxanthin, are among the set of 1O2 quenchers characterized by their high activity. An illustration of this can be seen in lycopene, which is produced as an intermediate in the metabolic process of carotenoids. It functions as an ally to nitric oxide, lipid peroxyl radicals, and reactive oxygen species (ROS), and may exert a protective effect against cancer, atherosclerosis, diabetes, and diseases associated with inflammation [99]. The extensive range of biological functions exhibited by phenolic compounds and conjugation of side chains to aromatic rings and the increased quantity of free hydroxyls have been linked to increased antioxidant activity [100].

Terpenoids constitute an additional extensive class of secondary metabolites found in plants. Antioxidant activity was observed in monoterpenes, sesquiterpenes, as well as diterpenes isolated from plants with aromatic compounds, according to in vitro tests [101]. Tiong et al. [102] reported on the hypoglycemic and antioxidant properties of vindolinine, vindoline, vindolidine, and vindolicine, all of which were extracted from the leaves of Catharanthus roseus. Furthermore, vindolicine decreases H2O2-induced oxidative injury to cells from the pancreas and exhibits the greatest level of antioxidant activity, suggesting that it may have possibility as a hypoglycemic agent.

Numerous studies have documented that resveratrol exhibits activity targeting the central nervous system (CNS). Despite traversing the blood-brain barrier (BBB), this element exhibits limited availability due to its rapid metabolism onto glucuronide and sulfate conjugates. Numerous lines of research suggest that in addition to other biologically significant antioxidant functions, therapeutic, and protective properties are also present [103]. In relation to the radical-scavenging action of resveratrol, computational and structural analyses indicate that the hydroxyl group located at the 4′-position is considerably more susceptible to oxidation during the antioxidant reaction compared to other hydroxyl groups. Resveratrol administered intraperitoneally has been found to enhance the activity of various indigenous antioxidant enzymes, including SOD and CAT, thereby inducing neuroprotective effects [104]. Resveratrol administration over an extended period of time ameliorates the cognitive decline induced by colchicine, decreases levels of MDA and nitrite, and replenishes depleted GSH.

Curcumin was shown to possess potent neuroprotective properties as an antioxidant by scavenging reactive oxygen species (ROS) and eliminating free radicals formed by nitric oxide (NO-) [105]. It is due to the H abstraction from these groups that curcumin possesses such exceptional antioxidant activity. Additionally, carbon-centered radicals and phenoxyl radicals are generated during the reactions of curcumin with free radicals at the methylene CH2 group. Further experimental evidence substantiating the antioxidant characteristics of curcumin was presented by utilizing an AD transgenic mouse model to illustrate how curcumin diminishes concentrations of proteins that have been oxidized in the brain that comprise carbonyl groups [106]. The antioxidant activity exhibited by curcumin in vivo might be facilitated by antioxidant enzymes including glutathione peroxidase (GSH-Px), superoxide dismutase (SOD), and catalase (CAT). It was recently established that curcumin operates as a Michael receiver via its interaction with glutathione (GSH) in the form of thioredoxin. An imperative marker of oxidative stress, which has been linked to the development of Alzheimer's disease, is a decrease in intracellular GSH concentrations. Additionally, curcumin has been found to augment the activities of antioxidant enzymes SOD and CAT in the striatum and midbrain of rodents injected with 1-methyl-4-phenyl-1,2,3,6-tetrahydropyridine (MPTP) [107]. Given the in vivo the results that peroxynitrite generates hyperphosphorylation, nitration, and formation of Alzheimer's-like tau, it has been documented that curcumin functions as a mediator in the direct detoxification of reactive nitrogen species, including peroxynitrite. As a result, curcumin exhibits antioxidant properties [108].

EGCG demonstrates protective effects beyond its anti-inflammatory properties through the regulation of various survival genes as well as the control of a multitude of antioxidant protecting enzymes. Neuronal toxicity that is linked to various neurodegenerative disorders is facilitated by sophisticated glycation end-products. By reducing ROS and MDA, EGCG enhanced SOD activity while safeguarding against glycation end products-induced neurotoxicity [109]. EGCG treatment increased SOD and GSH-Px activity in aged rodents treated with D-galactose while decreasing MDA levels in the hippocampus.Still, additional research is necessary to evaluate the risk associated with this method of administration. In addition, curative applications of curcumin have been assessed in the management of nonalcoholic fatty liver disorder, colitis with ulcers, chronic discomfort, cancer, premenstrual syndrome (PMS), as well as infection with Helicobacter pylori. [110] .The various disease conditions for which curcumin has been observed to provide benefits have been ascribed to its anti-inflammatory and antioxidant qualities [111].It suppresses the expression of COX-2 and iNOS, thereby preventing the harm caused by reactive oxygen and nitrogen species, and inhibits the signaling pathways IL-6, NF-κB, and MAPK, thereby decreasing astroglial activation, furthermore, it hinders the accumulation of tau and encourages the disassembly of tau oligomers in the laboratory [112]. Curcumin's prospective applicability in the treatment of Alzheimer's disease has generated considerable interest due to its antioxidant and anti-amyloid effects. Pomegranate juice along with extracts have been found to possess noteworthy bioactive properties, such as anti-inflammatory and antioxidant effects, in numerous animal and human studies [113]. Recent investigations on mice that analyzed the effects of extracts from pomegranate peels consumption revealed a reduction in pro-inflammatory cytokine expression, Aβ plaque density, and AChE activity, as well as an increase in neurotrophic factor, which is derived from the brain, expression [114]. Additionally, conjugated sucrose, fructose, and glucose, which are present in pomegranates, possess antioxidant effects, according to one research.

The flavonoid group of polyphenols comprises pigment compounds such as anthocyanidins and anthoxanthins, as well as flavans. Anthocyanidins, which are water-soluble compounds that are abundant in fruits like blueberries, possess strong anti-inflammatory and antioxidant characteristics [115]. Previous research has demonstrated that introducing blueberries into the diet of AD mouse models resulted in notable reductions in learning and memory deficits caused by the effects of oxidative stress and excitotoxicity, neuronal depletion, and AChE activity inhibition [116]. Anthoxanthins, comprising flavones and flavonols, are an additional category of flavonoid pigments. Fruits, vegetables, and botanicals contain the flavone luteolin, which possesses anti-inflammatory, antioxidant, antimicrobial, and neuroprotective properties, among others. Lutein inhibits the zinc-induced excessive phosphorylation of the tau protein at Ser262/365, as shown in laboratory investigations [117]. This is due to luteolin's antioxidant activity as well as ability to modulate the tau phosphatase/kinase system. Previous studies have demonstrated that EGb761 enhances dopaminergic transmission in the prefrontal cortex (PFC) of aged rats, stimulates the synthesis of a brain-derived neurotrophic factor, improves in vitro mitochondrial respiration, and inhibits the production of lipid peroxidation and superoxide free radicals in a rodent model of Parkinson's disorder [118]. EGb761 functions as an AChE inhibitor in addition to its robust antioxidant characteristics; consequently, many investigations have conducted comparisons between its clinical effects and those of pharmaceutical AChE inhibiting agents. The conventional method "anti-dementia treatments" include Ginkgo biloba, and their neuroprotective and antioxidant properties are highlighted [119].

### 5.3 Neuroprotective role of phytochemicals in AD

Flavonoids derived directly from the mulberry plant Morus alba are present in quercetin. Quercetin has medicinal value in preventing and treatment of various ailments, such as cardiovascular disease, cancer, and neurodegenerative disease, alongside may penetrate the blood-brain barrier [120]. In addition, quercetin has demonstrated characteristics that can inhibit the synthesis of histamine and stabilize the membranes of mast cells. Anti-inflammatory, anti-cancer, and anti-oxidant are some of its properties [121]. Quercetin has demonstrated efficacy as an inhibitor of various accumulation proteins, including Aβ, α-synuclein, and tau, in vitro. This is achieved through a direct association with misfolded proteins, which results in the maintenance of oligomeric species and the suppression of fibril development [121]. HT22 hippocampal neurons pre-treated with quercetin are resistant to oxidative damage and hyperphosphorylation of tau protein induced by okadaic acid. In a triple transgenic rodent model of Alzheimer's disease, additional research has shown that intraperitoneal administration of quercetin (25 mg/kg) decreased levels of Aβ and NFTs, ameliorated the neuroinflammatory procedure, and improved memory and cognitive impairment. In conclusion, quercetin is an excellent therapeutic agent for treating Alzheimer's disease and other neurodegenerative tauopathies, primarily because of its ability to traverse the blood-brain barrier.

Oxyresveratrol, a stilbenoid, is discovered in the heartwood of Artocarpus lakoocha, a member of the Moraceae family. Historically, it was utilized as the medicinal substance "Puag-Haad" [122]. In the past, oxyresveratrol was evaluated in relation to its anticancer properties, inhibitory activity against tyrosinase, and capacity to enhance the immune system. Oxyresveratrol is rapidly transported to tissues and has an availability of approximately 50 % [123]. Furthermore, treatment with oxyresveratrol prevented the phosphorylation of ERK, c-JNK, and p38 in cortical neuron cells produced with Aβ neurotoxicity, thereby directly inhibiting inflammation and apoptosis. Immune along with inflammatory responses, in addition to the development and release of proinflammatory cytokines such as interleukin-18 and IL-1β, are regulated by inflammasomes composed of NACHTLRR-PYD-containing proteins 3 (NLRP3). [124].

Resveratrol (3,5,40-trihydroxy-trans-stilbene) is a stilbenoid, a phytoalexin, and polyphenol derivative derived from Vitis vinifera. It is synthesized by a number of plants in reaction to infections or injury [125]. Resveratrol exhibits promising characteristics as a drug candidate, including cardioprotective, anti-carcinogenic, anti-inflammatory, and anti-carcinogenic properties [126]. Additionally, the ability of resveratrol to prevent hyperphosphorylation of the tau protein and/or facilitate dephosphorylation has been investigated. It is noteworthy that tau protein that has undergone hyperphosphorylation can bind resveratrol and become stabilized in a comparatively soluble state in a tau transgenic mouse model [127], thereby impeding tau aggregation into tangles. In laboratory models of severe brain injury, resveratrol supplementation immediately following the occurrence of traumatic brain injury decreases damage volume and oxidative stress. SIRT1, which is stimulated by resveratrol, reduces amyloid neuropathology in the brains of Tg2576 rodents and protects cells from Aβ-induced ROS production [128]. Given that resveratrol is a neuroprotective compound in the context of Alzheimer's disease, it is plausible to hypothesize that its antioxidant effects and SIRT1 activation may contribute to its ability to combat Aβ toxicity. Given the significant role that NF-kβ signaling activation plays in neurodegeneration, an additional correlation between Alzheimer's disease (AD) and the neuroprotective properties of resveratrol is its capacity to downregulate the expression of NF-kβ-modulated genes, including cathepsin, NO, prostaglandin E2 (PGE2), and iNOS. [129].

Rosmarinic acid, which is obtained from Melissa officinalis, has a long history of utilization due to its antioxidants, neurologically protective, as well as antioxidant characteristics [130]. Rosmarinic acid has the ability to inhibit inflammation and allergenic immunoglobulin reactions of polymorph nuclear leukocytes. Significantly, research has conclusively demonstrated that rosmarinic acid's anti-inflammatory, anti-apoptotic, and neuroprotective properties offer therapeutic advantages in the aftermath of brain injury [131]. Memory retention has also been observed to be enhanced by rosmarinic acid, possibly as a result of its experimental effects on increased BDNF levels and decreasing phospho-tau (p-tau) expression. In vitro studies have demonstrated that rosmarinic acid inhibits β-sheet assembly in the tau protein associated with Alzheimer's disease and decreases hyperphosphorylation of the tau protein [132]. Although rosmarinic acid possesses remarkable properties, more studies are required to validate its effectiveness against AD [133].

Quinic acid possesses anti-inflammatory and carcinogenic properties as well as is a cyclic polyol along with cyclohexane carboxylic acid [134]. An essential source of quinic acid derivatives is the Asteraceae family plant Aster scaber. Aster scaber ameliorates neurite growth by activating the TrkA signaling pathway, which is recognized as a crucial neurodegeneration process and whose significant anti-inflammatory effects in studies on animals make it a possible candidate for Alzheimer's disease. [135].

Curcumin, which is present in the condiment turmeric, originates from Curcuma longa, which belongs to the Zingiberaceae family of gingers. Curcuminoids, consisting of curcumin, demethoxycurcumin, and bisdemethoxycurcumin, are bioactive constituents found in turmeric. Experimental research and clinical research has demonstrated that these compounds have a multitude of beneficial effects [136]. Certain experts of healthcare administer turmeric intravenously, purportedly to address inflammatory conditions including psoriasis and joint discomfort. Curcumin, a substance that inhibits GSK-3β [137], is known to protect cells against tau-induced neurotoxicity. GSK-3β regulation of tau phosphorylation is facilitated by this enzyme. In recent studies involving rodents and rats, the potential of curcumin to mitigate inflammation and mitochondrial dysfunction in models of neurological insult has been investigated. Curcumin decreased post-injury lesion diameters and inflammatory biomarkers in brain tissue, and enhanced mitochondrial function and behavioral outcomes, according to the findings [138]. Furthermore, a transgenic mouse study revealed elevated levels of damaging DNA in comparison to the control group of mice, and adding curcumin to the diet was found to substantially mitigate the damage. Curcumin exhibits significant antimicrobial properties that might exert either direct or indirect impacts on the accumulation of A or other neuropathological pathways that contribute to the development of AD. Curcumin's antioxidant and anti-amyloid properties have sparked considerable interest in its potential therapeutic applications for AD. The insoluble nature of curcumin in water, nevertheless, has limited its application. By synthesizing curcumin molecules that retain their anti-oxidative attributes, are actually non-cytotoxic, and have the potential of destroying amyloid aggregates, this limitation may be surmounted, allowing for an integrated strategy to the prevention of Alzheimer's disease

Zingiber officinale, frequently referred to as ginger, is a member of the Zingiberaceae family and has been utilized historically for its aromatic and condiment properties. 6-Shogaol is the primary active component of Zingiber officinale. Scholarly investigations have demonstrated that shogaol (6-, 8-, and 10-shogaol) possesses potent anti-inflammatory and anti-oxidant characteristics [139]. Furthermore, a multitude of in vitro investigations have substantiated the protective properties of 6-Shogaol against neurodegenerative disorders, most notably Alzheimer's disease, through its ability to improve memory, suppress inflammation, and stimulate the antioxidant system. Furthermore, the 6-shogaol extract was found to increase BDNF expression and decrease iNOS, NF-kB, and COX-2 levels, all of which are crucial components of the neurodegeneration process. The phytotherapeutic potential of 6-shagaol in the treatment of neurodegenerative disorders such as Alzheimer's disease was uncovered by these investigations.

Citrus fruits, including oranges (Citrus sinensis) along with grapefruit (Cirus paradis) are abundant in the potent antioxidant naringenin [140]. The potential of naringenin's antioxidant, anti-inflammatory, as well as neuroprotective properties to enhance memory function in individuals with diabetes type 2 and dementia has been extensively documented [141]. Naringenin treatment induces a reduction in the expression of nucleotide oligomerization domain protein 2 (NOD2) and NF-kB in a rodent model of injury involving serious brain injury.

Delphinidin is an anthocyanidin-class dye that is accessible in water. Red wine as well as berries, which contain noteworthy pharmacological activities including anti-inflammatory as well as antioxidant qualities, are profuse in this substance. In relation with the neuroprotective properties of delphinidin, extant research indicates that it impedes the hyperphosphorylation of tau as well as the activation of GSK-3β in PC12 cells, both of which are induced by Aβ [142]. In a similar manner, it inhibits calcium levels within cells to avoid neurodegeneration induced by Aβ.

Epigallocatechin-3-gallate (EGCG) is a flavonol predominantly present in green tea leaves. There has antioxidant, antitumoral, antibacterial, and neuroprotective properties have garnered significant attention in both laboratory settings [121]. EGCG binds to a partially misfolded intermediate, which prevents seeding and rescuing cells from tau-induced toxicity, in accordance with new research [121]. Nevertheless, EGCG is a phytochemical that has the ability to increase the breakdown of phosphorylated tau molecules in neurons in a selective manner; this may have substantial implications for preventing the progression of AD. The pace at which EGCG crosses the BBB is minimal, and its bioavailability was estimated to be around 5 % following oral administration. It is important to acknowledge that rat hippocampal neurons perished in response to high doses of EGCG via the mitochondrial-dependent process. Furthermore, EGCG exhibits prooxidant and proapoptotic properties at elevated levels [143]. Furthermore, the possibility that inhibiting monoamine oxidase (MAO) activity could provide protection against oxidative neurodegeneration is intriguing. The intake of adult rat brains with EGCG inhibited the activity of MAO-B, thereby preventing physiological peroxidation. In a recombinant AD rodent model, intraperitoneal EGCG injection reduced brain Aβ neuropathology and enhanced cognitive function [144]. EGCG specifically hinders the fibrillogenesis of Aβ by binding to natively stretched polypeptides and obstructing their transformation to intermediates of toxic formations.

Morin, a flavonol, is found in dyer's mulberry, guava, and imitation orange, among other fruits and vegetables. It functions as an anti-inflammatory agent, antioxidant, hepatoprotectant, as well as antihypertensive. Morin may prevent GSK3β-induced tau phosphorylation in vitro as well as inhibit GSK3β activity as a neuroprotective agent [145]. Furthermore, morin administration significantly reduced tau hyperphosphorylation in APPswe/PS1dE9 double transgenic mice by inhibiting the CDK5 signaling pathway. Meganatural-az (MN) represents a collection of grape seed-derived polyphenolic formulations. According to a rodent model of Alzheimer's disease [146], it decreases Aβ oligomerization and enhances memory. Additionally, MN facilitates tau oligomer breakdown. The polyphenol oleocanthal is extracted from excess virgin olive oil. Its nonsteroidal anti-inflammatory activity was found to be comparable to that of ibuprofen. The polyphenol in question exhibits an inhibitory impact on the fibrillation of both Aβ and tau [147]. With respect to the oligomerization of tau, oleocanthal inhibits the misfolding of tau and preserves its state of unfolded nature. Therefore, this polyphenol warrants further investigation as a possible substance to incorporate into innovative treatments for neurodegenerative tau disorders.

## 6 Disease modifying therapies of Alzheimer's disease

The highly anticipated era of disease-modifying medication for Alzheimer's disease has already begun and will significantly influence the perception and management of the condition (Ono et al., 2020). However, these novel therapies will present hurdles in ensuring fair and equal access. The medications that are most likely to be widely used in clinical practice are lecanemab and donanemab. Lecanemab received complete approval from the FDA on July 6, 2023, making it the first medicine for Alzheimer's disease that can affect the progression of the illness. Lecanemab is a specific kind of antibody that is designed to target and bind to amyloid-β soluble protofibrils and oligomers in humans. It has a strong attraction to these forms of amyloid-β, but a less attraction to amyloid-β monomers and insoluble fibrils [148]. The original FDA rapid clearance for donanemab was denied in January 2023, based on the findings of a phase 2 study. However, on February 7, 2024, it was subsequently granted permission [149]. Patients who suffer from moderate cognitive impairment (MCI) or mild Alzheimer's disease may benefit from these intravenous monoclonal antibody treatments since they are able to pass the blood–brain barrier and eliminate cerebral amyloid-β (Aβ) [150]. The introduction of disease-modifying medications for Alzheimer's disease might lead to an increase in the number of persons seeking clinical care. This includes those already diagnosed with the illness, those with various forms of dementia, and people who are worried about their chances of getting dementia [151]. The difficulties associated with planning for the prompt and fair distribution of disease-modifying medicines for Alzheimer's disease are significant. New therapies are expected to first be implemented at specialised facilities. However, as knowledge and expertise are acquired, these medications will need to be made available to all eligible individuals. These disease-modifying therapies for Alzheimer's disease present a significant challenge, but they also present an unparalleled chance to alter the way dementia is understood, enhance clinical pathways and patient care for all patients, and set the stage for the delivery of the upcoming generation of therapies as they become available [152].

## 7 Conclusion and outlook

AD has a multilateral pathogenesis, as well as the development of novel therapies capable of targeting different targets in brain, is necessary for managing its polyetiological origin, cognition and motor dysfunction, melancholy, and neurodegeneration. Phytochemical entities capable of targeting specific targets implicated in the pathogenesis of AD are described in this article. Furthermore, while the previously mentioned phytocompounds' neurological protective effects seem promising due to their diverse biological activities, at the very least, supplementary long-term research is required to determine whether or not they delay the progression of AD. In summary, it has been demonstrated that polyphenols possess significant potential as protective compounds within the realm of neuroscience, and this knowledge continues to grow. In conclusion, pertinent findings regarding the medical advantages of phytochemicals in neurodegenerative disease have been discussed in this review. Further investigation is warranted to ascertain whether these phytochemicals alleviate symptoms in patients diagnosed with Alzheimer's disease (AD), as well as to determine whether the anti-AD, neuroprotective, and anti-inflammatory properties documented in this study are also associated with a deceleration of the neurodegeneration process in AS. Further preclinical investigations are warranted to examine the impact of phytochemicals on neurodegenerative disorders, with a particular focus on Alzheimer's disease, so as to increase our understanding of the protective mechanisms exhibited by phytochemicals in such conditions. Moreover, it is imperative that scientific investigations incorporate the therapeutic advantages of natural products, given the ample body of literature that consistently affirms their effectiveness and safety when employed in lieu of conventional therapies. In-vitro and in-vivo studies have consistently indicated that phenolic-derived phytochemicals exhibit more potent anti-inflammatory properties and target subsequent signaling pathways, which bodes well for the prevention of Alzheimer's disease development.

## Author contribution

RAN, RR, RKK, AS and SKS conceptualized and designed the first blueprint for the manuscript. RAN, RB, AFA, AKJ, HF, AOB, AH, WFA, ASA, MA, AA, AS and SKS collaborated on the paper's composition and opinion, as well as making important edits and signing off on the final version of the manuscript. All authors have read and approved this draft for publication.

Data Availability Statement: Data sharing does not apply to this article as no new data were created or analyzed in this study.

## Declaration of competing interest

The authors state that they have no competing interests in this article.
